# Autism Children and English Vocabulary Learning: A Qualitative Inquiry of the Challenges They Face in Their English Vocabulary Learning Journey

**DOI:** 10.3390/children9050628

**Published:** 2022-04-28

**Authors:** Haida Umiera Hashim, Melor Md Yunus, Helmi Norman

**Affiliations:** 1 English and Linguistics Department, Academy of Language Studies, Universiti Teknologi Mara (UiTM Shah Alam), Shah Alam 40450, Malaysia; haidaumiera@gmail.com; 2 Faculty of Education, Universiti Kebangsaan Malaysia (UKM), Bangi 43600, Malaysia; helmi.norman@ukm.edu.my

**Keywords:** autism education, challenges, ESL learning, language learning, vocabulary learning

## Abstract

Language learning is one of the most important aspects of an individual’s life development and growth. Normal learners’ English language acquisition differs depending on their preferences and learning styles. Children with autism, on the other hand, appear to have different learning methods and routines. There has been a lot of previous work on the language learning styles of normal learners, but not much on the language learning path of children with autism. Due to that, this study aims at investigating the challenges children with autism face in their English vocabulary learning. Observations and focus group discussions were conducted with 45 children with autism, 4 autism education teachers, 2 occupational therapists and a parent of children with mild autism children to investigate the challenges autism children face in their vocabulary learning. The findings have gathered that autistic children encounter a few challenges occasionally in their English vocabulary learning journey. The data were gathered and arranged thematically. Further recommendations and suggestions are discussed in this study.

## 1. Introduction

Autism spectrum disorder (ASD) causes a child to have chronic difficulty with social communication and interactions in a variety of settings, as well as to exhibit confined, repetitive patterns of behaviour, interests, and hobbies [1,2,3]. There is a pressing need for learning to be inclusive of all children, particularly those with autism spectrum disorders (ASD), as we work toward UNESCO’s sustainable development objectives (number four of which emphasises inclusion in education through excellent education).

Despite their challenges, children with autism have the right to participate in all parts of their lives, including learning English. As a result of technology breakthroughs and the world of the 4th Industrial Revolution [4,5], English has become a global language, and inclusive education will help children with autism survive in the future [6]. Learning a language is a process, and the first stage is to learn vocabulary. Word knowledge, or vocabulary, is the foundation for most aspects of language and achievement, according to [5]. According to [7], vocabulary development is an important part of language learning.

According to the literature, several researchers have explored possibilities and devised techniques to help autistic youngsters with their language skills. Take the use of technology as an assistive device for autistic children, for example. Each autistic child is on his or her own. Autism affects children all around the world, and they share comparable concerns and challenges as a result of their condition [8]. Each autistic child has unique learning styles and preferences, which can be both beneficial and challenging for them as students. For autistic children, however, learning a language is a difficult undertaking [9,10].

The four primary talents covered in language studies are listening, reading, speaking and writing. A language cannot be mastered without first mastering its vocabulary [11]. The acquisition of vocabulary is an important part of the learning process, especially in English language learning. Beginning in kindergarten and continuing through university education, the language is taught in schools [12]. Having English language skills, whether for generally developing or autistic children, can aid them in the future with their schooling and employment. However, understanding the English language will be almost impossible if they do not understand the vocabulary component of the language [13,14]. In conjunction to this, it is believed that there is a need for a study to investigate and analyse the challenges faced by children with autism in their English vocabulary, as well as their needs to help them to have a smooth sailing English vocabulary learning journey.

## 2. Literature Review

Many parents of autistic children wonder if speaking to their children in several languages is a bad idea. Some parents may not want to stress their autistic child by exposing him or her to several languages because ASDs usually involve linguistic issues. Without fully understanding current facts, some general practitioners have urged that parents of autistic children speak English rather than their local language in a country where English is the official language. There is no evidence, however, that introducing a child with autism to a second language will prevent them from acquiring it. In fact, studies suggest that limiting a child’s exposure to his or her native tongue makes it far more difficult for the child to socialise, even within his or her own family. Parents keep their children out of conversations by not speaking in their native tongue. Autistic children can have social issues and are more likely than their peers to catch up on their parents’ language. It may be difficult for parents who do not speak a second language well to communicate with their autistic children. If these parents choose to communicate in English as their primary language, they may have additional linguistic challenges. A literal comprehension of words is usually associated with autism. Understanding colloquialisms and idiomatic English may be difficult for autistic language learners.

The process of language acquisition starts at birth. Internalisation, or the application of sounds to pictures, is the primary method through which a child learns language. When a child understands a language’s grammar, they progress from internalisation to interpretation. By the conclusion of the first 48 months, the child will have developed rudimentary language skills and a vocabulary that is mostly limited to their own experiences [15,16]. Over development, the child expands their lexicon and employs increasingly complex sentence structuring in their utterances, building on this foundational knowledge of language structure. Language is the primary means of transmitting social and cultural norms, as well as history and behaviour, from one generation to the next. Patients with autism spectrum disorders (ASD) usually lack the communicative skills that their TD peers possess. Is there a ‘theory of mind’ in the autistic child? Due to their neurologically diminished social aptitude, an autistic child’s language development is impeded by a lack of proper social interaction and, as a result, social interpretation. To varying degrees, this has an impact on the key language domains of discourse, pragmatic functions, and grammar. As a result, autistic people often have a limited vocabulary and syntax, strange tone or pitch in their speech, and little or no grasp of how to carry on a conversation.

An autistic child’s language development is hampered by a lack of proper social engagement and, as a result, social interpretation due to their neurologically decreased social aptitude. This has an impact on the key language domains of discourse, pragmatic functions, and grammar to varied degrees. As a result, autistic people frequently have a limited vocabulary and syntax, unusual tone or pitch in their speech, and little or no understanding of how to converse. Autistic people’s language development is frequently delayed and hampered as a result of their neurological illness. A child’s lack of age-appropriate verbal development could indicate that he or she has autism. Some severely autistic children never learn to communicate. Theory of Mind explains why ASD children lack or have limited verbal and social skills (ToM). They also use ToM to show how linguistic talents, such as reading and writing, which are not centred on social interaction or interpretation, are preserved.

How to treat children with special needs, such as those with autism spectrum disorder [17], so that they can learn and receive a proper education [18,19] is one of the most difficult challenges for teachers and families. Many autistic children have not matured as expected, necessitating special education to fulfil their unique demands [20] According to inclusive education theories, all students, including those with special needs, should be provided with the same components of learning subjects, with varying compositions based on the students’ abilities and requirements [21,22]. English is no exception, as previously indicated. Teachers are responsible for assisting students with special needs in strengthening their English communicative skills so that they can participate in English conversation as well. In this study, we concentrate our efforts on English teaching practises for students with special needs, particularly children with ASD. Data collection and consultations are used to assess each student’s unique learning profile while developing educational programmes for students with ASD.

## 3. Methodology

This study employed a qualitative methodology of research in which field notes and focus group discussions were used as instruments. Forty-five children with autism, 4 autism education teachers, 2 occupational therapists and 1 parent of mild autism children were involved in this study. The respondents were purposely chosen for their leverage as having a wider perspective on the challenges children with autism face and their needs in their English vocabulary learning. The findings gathered from the field notes and focus group discussion sessions were analysed, and the Cohen Kappa value is gathered, then thematically arranged into a subsection of a. The challenges autism children face in their English vocabulary learning.

Cohen Kappa value analysis was performed to measure the degree of expert or evaluator agreement on the built topic in order to determine the trustworthiness of qualitative data [23]. Braun and Clarke (2019) state that evaluator agreement is critical in determining the high reliability of each unit utilised to characterise a theme. The data was gathered and matched with supporting data collected through document analysis as reinforcement after all interview transcripts were validated by study participants. After that, the data was carefully collated to determine the study’s theme. Once the theme findings are obtained, the data coding process is completed. Then, as external assessors, a set of expert consent forms were created to examine the level of expert consent and to test the sustainability of the researcher’s theme construction.

The qualitative data in this study’s qualitative data can be verified by professionals in the qualitative and content fields, namely TESL and Special Education. To calculate the Cohen Kappa Value, researchers solicited the help of two experts in respective domains. The Cohen Kappa method was used to further examine the expert consent form’s findings in order to estimate its index level [24]. Table 1 shows the list of experts who participated in the theme agreement analysis.

After all of the experts had completed their assessments, the researcher calculated the agreement’s value. Each expert evaluator’s coefficient of agreement was recorded separately and then added together to get a total coefficient of agreement for the entire process. To determine the level of Cohen Kappa agreement, the values recommended by Landis and Koch (1977) [25] were used by the researcher as shown in Table 2.

The results of the calculation of the Cohen Kappa reliability value have enabled the researcher to obtain the average value of Cohen Kappa as shown in Table 3 and Table 4 below:

The Cohen Kappa (K) agreement value for this study was 1.0, indicating that the qualitative data was reliable. This is a fantastic value for dependability [25]. In other words, the researcher’s unit of analysis corresponds to the theme given. The data gathering processes are discussed in the next section of the methodology.

## 4. Findings

The findings of this study were gathered, analysed, then thematically arranged into emerging themes to discuss the challenges autism children face in their English vocabulary learning journey. The data is presented below.

### 4.1. Different Characteristics and Preferences

Each child with autism has specific traits, and each of them reacts or responds differently to external exposure or new things. Each of the autistic youngsters, according to OT1, has unique qualities and preferences.

“each one of them has different characteristics…”

(FGD, OT1)

Table 4 displays respondents’ responses and agreements on the different characteristics and preferences as one of the challenges for autism children. OT1 noted that each child with autism may have a unique condition. Some people are drawn to bright colours and find them fascinating. Some people, on the other hand, may find muted hues to be more relaxing and brilliant colours to be distressing. This was later backed up by OT2, who stated that children with autism do differ from one another.

“some might be sensitive towards sounds, lights, and touch…”

(FGD, OT2)

“some are very attracted to bright and vibrant colours…”

(FGD, OT2)

The data from the field notes also support the assertions made during the focus group discussion sessions by both occupational therapists. During observations with autism youngsters at the autism centre, it was discovered that some of them were interested by and drawn to brilliant colours. Furthermore, their differing preferences can be demonstrated in terms of the incentive they prefer once the lessons are completed. Each child prefers a particular type of incentive. The ability to rest for 10 min may appeal to one child, but it may serve as a negative reinforcement for the other. Receiving cookies can be a treat for one child, while having to eat can be a punishment for another. These scenarios demonstrate that each autistic child has unique qualities and interests.

Furthermore, during the focus group discussion, both occupational therapists agreed that predicting their behaviours and reactions to external stimuli is difficult. OT1 went on to say that each of them reacts and responds differently to external stimuli and exposure due to their variances in preferences and characteristics. Both instructors and occupational therapists agreed that being introduced to new people, surroundings or routines might cause them to behave differently, and at times can drive them to throw tantrums.

“each one of them reacts and responds differently towards external exposure…”

(FGD, OT1)

Due to the aforementioned problem, OT2 believes that it is critical for family members to comprehend and be aware of each of the child’s traits, including preferences, because they play an important role in the language learning journey of autistic children.

“it is crucial to be alert to each learner’s characteristics…”

(FGD, OT2)

Furthermore, throughout the observations, it was seen that each child has their own set of learning resources, such as books and workbooks. The researcher has gotten a lot of attention as a result of this circumstance. During a focus group discussion with autism education instructors, occupational therapists and parents, it was discovered that customised learning materials are used to accommodate to the variances in traits and preferences among autism children.

“each one of them has their own learning materials…”

(FGD, T1) + (FN)

According to autism education professionals, having their own learning materials is critical because they do not like to share. The difficulties that autistic children have in learning English vocabulary are exacerbated by their various features and preferences.

### 4.2. Different Levels of Cognitive Abilities

During observations conducted at the autism centre, it was discovered that each child has their unique set of learning objectives. T1 also brought up this topic during the focus group sessions.

“each one of them has their own learning objectives…”

(FGD, T1) + (FN)

The maximal learning objectives for moderate autistic youngsters, according to the researcher, are 11 and varied from one another. On the other hand, it has been discovered that the maximum learning objectives for severe autistic children are eight and that they differ. As a result of seeing and understanding this, the researcher discovered that each of the autism children has their unique learning objectives due to their differing cognitive capacities. T3 believes that the disparities in cognitive capacities across autism children make it difficult to assess their progress.

“it is crucial for ASD children to have distinctive learning objectives due to their differences in abilities…”

(FGD, T3)

Table 5 above displays respondents’ responses and agreements on the differences of cognitive levels of abilities among children with autism.The fact that children with autism have varying levels of cognitive skills adds to the difficulties they encounter in learning English vocabulary, as it is impossible for teachers to generalise their teaching and lessons to accommodate all levels of cognitive capacities.

### 4.3. Visual Strategy Learners

In addition to the problems listed previously, another challenge noted by autism children in their English vocabulary learning is their preferred learning style. Visual approach learners, such as children with autism, prefer to learn through pictures and images. Because they do not have images or painted in their heads, it is difficult for them to understand what they are learning. Teachers, who spend most of their time with youngsters were the most enthusiastic about this. They believed that pictures were the best way for autistic children to learn.

“these children are mostly visual strategy learners…”

(FGD, T3)

“they tend to learn better through pictures and images…”

(FGD, T4)

Occupational therapists subsequently backed up the preceding remarks by mentioning the usage of “social story.” Because children learn better through visuals and pictures, the use of ‘social story’ is becoming more common in autism education.

“we use social stories most of the time…”

(FGD, T1)

During the focus group session, another mom agreed that her child learns best through appealing movies and graphics. If people with autism are not included in their learning process, they may struggle to understand what they are learning and lose attention.

Table 6 displays respondents’ responses and agreements on how usually children with autism are associated with being a visual strategy type of learners. Being a visual approach type or learner is often a barrier for autistic children when learning English language. Because they are unable to learn and acquire language in the same way that other typically developing youngsters do through traditional vocabulary learning, they find it difficult to learn vocabulary.

### 4.4. Short Retention Power

The data also revealed that children with autism frequently have low retention power. Autism children learn through routines and repetitions, according to both teachers and occupational therapists.

“ASD children learn through repetitions…”

(FGD, T2)

Autism children, according to T2, learn through repetition. In order to understand a lesson, they must repeat the same tasks or practises several times. Teachers are then required to repeat the same lecture using the same materials, which, according to T2, can be exhausting for teachers. Children with autism are often recognised for their repetitive actions. T2 went on to say that children with autism dislike it when their daily routines are changed or tweaked because they are unable to be flexible because they learn through routines.

“ASD children usually practice learning by routines…”

(FGD, T2)

When the topic came up during the focus group discussion, T1 interjected and stated that while it is possible to introduce new lessons or routines to autism children, due to their short retention power, a proper introduction to the lessons must be conducted and repeated over and over before the lessons can be implemented. T1 noted that autism youngsters listen to instructions and will follow them; nevertheless, when it comes to introducing them to new stimuli or exposure that is unfamiliar to them, a good introduction is required.

“these children do listen to instructions, but a proper introduction is very much needed every time…”

(FGD, T1)

The identical comments came from the parent, who stated that her child’s daily routine is the same. His routines should be the same every day, and no tiny changes should be made without prior approval, or he may throw tantrums. The topic matter is confirmed correct when the learning routines and activities supplied for the autism youngsters are the same every day during observations. Their day will begin with morning classes, followed by rest, lunch, and circle time (or “playtime”) after lunch. The children at the autism centre are all familiar with their routines and what to expect later. According to the teachers, the activities cannot be swapped out. Table 7 displays respondents’ responses and agreements on having short retention power among children with autism is to be identified as one of the challenges for them to acquire English vocabulary.

Having low retention power as one of the traits within autism children, parents and teachers are forced to repeat the same routines and lessons day after day. It can be exhausting for teachers to repeat the same teachings every day to ensure that pupils understand them, and the same can be said for parents at home. As a result, this aspect is one of the contributing elements to the difficulties that autistic children have learning English vocabulary.

### 4.5. Restricted Behaviors

Autistic children’s confined and repetitive activities can be noticed as a result of their short retention power and cognitive difficulties. OT1 said during the focus group discussion that the behaviours of autistic children are fairly limited. The effect of having the same and repetitive routines every day has taken its toll on the behaviours of autism youngsters, according to OT2. They are so accustomed to the same daily routines that they are unable to broaden their actions in order to grow, such as connecting with others, initiating conversations, and so on.

“ASD children usually have a set of repetitive and restricted behaviours…”

(FGD, OT1)

The effect of having the same and repetitive routines every day has taken its toll on the behaviours of autism youngsters, according to OT2. They are so accustomed to the same daily routines that they are unable to broaden their actions in order to grow, such as connecting with others, initiating conversations, and so on.

“students with autism usually have trouble reacting to and initiating social relationships…”

(FGD, OT2)

Autistic children are unable to acquire additional language due to their inability to react and start social relationships, as a result of the same daily patterns that lead to their confined and repetitive behaviours. A limited vocabulary results from a lack of communicating and starting discussions. Table 8 displays respondents’ responses and agreements on how restricted behaviours can contribute to one of the challenges that hinder autism children to acquire English vocabulary.

A limited vocabulary results from a lack of communicating and starting discussions. As a result, one of the obstacles autistic children experience in learning English vocabulary is the nature of having repetitive and restricted activities.

### 4.6. Harder to Grasp More than 3-Syllable Words

The situation involving autism youngsters not being able to grasp more than 3-syllable words is the last but not least challenge that came from the research. During the focus group discussion, a parent of an autistic child stated that her child understands English words more quickly and accurately than Malay terms. The reason for this is that her child has stated that he prefers English terms than Malay words because they are simpler to pronounce. According to the parent’s comment, her autistic child finds English words simpler to speak because of the quantity of syllables. He believes English words to be easier because they are simpler and have fewer syllables in each word. T1 acknowledged that ASD children do have a tendency to learn English faster than their mother tongue language after seeing this. OT1 went on to say that because of the linguistic component of the language, autistic youngsters prefer and absorb English language more quickly.

“learners with autism have a tendency for picking up English language faster than their mother tongue language…”

(FGD, T1)

“learners with autism will absorb English words and language more quickly due to the linguistic component of the language…”

(FGD, OT1)

It has been demonstrated that this is one of the reasons why autistic children struggle to learn and acquire new words. It is more difficult for children to comprehend words with more than three syllables, thus providing them with a platform to improve their English vocabulary acquisition could aid in their language development and articulation. Table 9 displays respondents’ responses and agreements on autism children find it harder for them to grasp more than 3-syllable words as one of the challenges for them to acquire English vocabulary.

## 5. Discussion

The findings revealed that children with autism suffer certain difficulties in learning English vocabulary. The unique traits that children with mild autism possess, as well as the restriction in terms of teaching and learning materials for them to learn English vocabulary, are two of the most significant obstacles that prevent them from having a smooth English vocabulary learning experience. Each child with autism is unique and has their own set of features. In their paper characterising children with autism in general, [5] noted the qualities and characteristics of autistic children. In terms of taste and passion, they are not the same. As a result, when it comes to catering to children with autism during the teaching and learning process, generalisation is not an option. In their study, [2] agreed that each child with autism is unique, and it is very difficult to generalise autism or children with autism. Furthermore, children with autism use visual strategies to learn. Images and visual aids help them study more effectively. Social tales are a visual learning and teaching aid that is frequently used by educators educating children with autism, and the word ‘social stories’ has become a widespread term used in the autism education profession.

Children with autism, unlike ordinarily developing children, tend to repeat actions and learn through patterns. Their daily routines should be the same, and if anything changes, they will either throw tantrums or become puzzled by the changes because they are not used to them. This is demonstrated by the data acquired. They will perform the same action again and over again when they discover new things. As a result of all of these qualities and traits, children with autism have the latter obstacle in learning English language. Because of their individuality, children with autism suffer limitations in terms of the learning materials they can use to improve their English vocabulary. Teachers find it difficult to consider designing distinct resources for each of the children in the classroom throughout the teaching and learning session because they all have different traits and preferences. Although visual aids can occasionally be useful in attracting a child’s attention, other children find them upsetting and uninteresting since the colour or style of the materials may not be to their liking.

Autistic students typically require their own learning materials because they do not like to share due to their unique traits. At times, teachers must create materials by hand to cater to each child in order to capture their interest and keep them engaged in the English vocabulary learning session in the classroom. Handmade materials do not survive long, and all of the teaching and learning tools at the autistic centre are already worn out, according to observers. Children with autism have trouble learning and acquiring vocabulary as a result of their cognitive impairments. Despite prior studies showing that learners with autism grasp English language faster than their mother tongue due to the linguistic component of English, findings demonstrate that children with autism still struggle with the language. Autism has been shown to be different and unique in its own manner in children. They each have distinct traits and characteristics that set them apart from one another. Due to their uniqueness, each autistic child has his own learning style and preference. In their study, Hashim et al. [14] proved that children with autism encounter difficulties and concerns in their language learning. Due to their various habits and traits, teachers usually struggle to personalise learning materials for their students.

## 6. Limitations and Delimitations of This Study

This study’s participants were chosen with the features of children with moderate autism in mind. Youngsters with mild autism, commonly known as high functioning children, were specifically chosen because they have fewer severe autism symptoms. Because the researcher does not have a background in Special Education, he or she may struggle to meet the study’s objectives due to a lack of expertise working with children with severe autism. Due to permission considerations provided by the learners’ parents, as well as their conditions and actions, the researcher was unable to undertake a random sampling of participants. The special education teachers in the evaluation phase were also hand-picked for their experience working with autistic youngsters.

## 7. Conclusions

The findings revealed that children with autism suffer certain difficulties in learning English vocabulary. The unique traits that children with mild autism possess for being autistic, as well as the restriction in terms of teaching and learning materials for them to learn English vocabulary, are two of the most significant obstacles that prevent them from having a smooth English vocabulary learning experience. Each child with autism is unique and has their own set of features. In terms of tastes and passion, they are not the same. As a result, when it comes to catering to children with autism during the teaching and learning process, generalisation is not an option. Past studies have agreed that each child with autism is unique, and it is very difficult to generalise autism or children with autism. Furthermore, children with autism use visual strategies to learn. Images and visual aids help them study more effectively. Social tales are a visual learning and teaching aid that is frequently used by educators educating children with autism, and the word ‘social stories’ has become a widespread term used in the autism education profession.

As agreed by past literature, children with autism, unlike ordinarily developing children, tend to repeat actions and learn through patterns. Their daily routines should be the same, and if anything changes, they will either throw tantrums or become puzzled by the changes because they are not used to them. This is demonstrated by the findings, which reveal that as they learn new things, they will do the same thing again and over again. As a result of all of these qualities and traits, children with autism encounter the latter issue in their English vocabulary development. Because of their individuality, children with autism suffer limitations in terms of the learning materials they can use to improve their English vocabulary. Teachers find it difficult to consider designing distinct resources for each of the children in the classroom throughout the teaching and learning session because they all have different traits and preferences. Although visual aids can occasionally be useful in attracting a child’s attention, other children find them upsetting and uninteresting since the colour or style of the materials may not be to their liking.

Autistic students typically require their own learning materials because they do not like to share due to their unique traits. At times, teachers must create materials by hand to cater to each child in order to capture their interest and keep them engaged in the English vocabulary learning session in the classroom. Past literature has investigated the factors that hinder children with autism from communicating properly, and this study intends to further contribute to the findings by providing findings of challenges, as well as factors that autistic children encounter, which hinder them from learning vocabulary.

As a result of the obstacles that children with autism encounter, gathered in this study, it is hoped that related parties in the field of education could benefit from the findings. Moving towards the aim of inclusive education involving learners with disabilities, the findings gathered in this are hoped to provide an insight into the challenges that related parties could take into consideration for future policy making. The researchers believe that learning materials that can also be used as self-paced learning materials for children with autism are needed. Nonetheless, the content should be able to accommodate the features of autistic children and solve the difficulties they have in learning English vocabulary, in order to bridge the gap between typically developed learners and autism learners.

## Figures and Tables

**Table 1:** List of theme agreement analysis experts.

No.	Position	Field	Institution
1.	Senior Lecturer	TESL	Universiti Teknologi Mara (UiTM)
2.	Senior Lecturer	Special Education	Universiti Kebangsaan Malaysia

**Table 2:** The interpretation of the Cohen Kappa value.

Cohen Kappa Value	Interpretation
Less than 0	Very weak
0.01–0.20	Weak
0.21–0.40	Quite weak
0.41–0.60	Moderate
0.61–0.80	Good
0.81–1.00	Very good

**Table 3:** The average value of Cohen Kappa for the challenges.

Challenges autism children face in their English vocabulary learning journey	Expert 1	Expert 2	Average Value
	23 − 11.5 _____ = 1.0 23 − 11.5	23 − 11.5 _____ = 1.0 23 − 11.5	1.0 + 1.0 ______ = 1.0 2

**Table 4:** Different characteristics and preferences of autism children.

Challenges	T1	T2	T3	T4	OT1	OT2	P
Different characteristics and preferences	x	x	x	x	x	x	x

Sign ‘x’ indicates the same responses gotten from all participants.

**Table 5:** Different levels of cognitive abilities of autism children.

Challenges	T1	T2	T3	T4	OT1	OT2	P
Different levels of cognitive abilities	x	x	x	x	x	x	-

Sign ‘x’ indicates the same responses gotten from all participants.

**Table 6:** Visual strategy type of learners.

Challenges	T1	T2	T3	T4	OT1	OT2	P
Visual strategy type of learners	x	x	x	x	x	x	x

Sign ‘x’ indicates the same responses gotten from all participants.

**Table 7:** Short retention power.

Challenges	T1	T2	T3	T4	OT1	OT2	P
Short retention power	x	x	x	x	x	x	x

Sign ‘x’ indicates the same responses gotten from all participants.

**Table 8:** Restricted behaviours.

Challenges	T1	T2	T3	T4	OT1	OT2	P
Restricted behaviours	x	x	x	x	x	x	-

Sign ‘x’ indicates the same responses gotten from all participants.

**Table 9:** Harder to grasp more than 3-syllable words.

Challenges	T1	T2	T3	T4	OT1	OT2	P
Harder to grasp more than 3-syllable words	x	x	x	x	x	x	-

Sign ‘x’ indicates the same responses gotten from all participants.

## Data Availability

The data presented in this study is openly available.
